# Supplementary material for: Using visual attention estimation on videos for automated prediction of autism spectrum disorder and symptom severity in preschool children
Source: PLoS One. 2024 Feb 12;19(2):e0282818. doi: 10.1371/journal.pone.0282818 (PMC10861059; doi:10.1371/journal.pone.0282818)
Supplement: Attachment — Submitted filename: Response_to_Reviewers.docx [file pone.0282818.s001.docx]

Dear Dr. Antoine Coutrot,

Thank you very much for sending the three reviewers’ comments on our manuscript. We thank you and the reviewers for taking their valuable time in reviewing our manuscript. We are very pleased to see that all reviewers found our manuscript to be valuable and/or suggested further improvements to our work. We agree with all the points raised and made revisions accordingly. We believe that they have greatly improved our submission.

We also thank you for providing us with an opportunity to submit a revision and clarify any concerns raised by the reviewers. To address these concerns, we made the following:

Major changes:

1. We have expanded the Related Works section with the inclusion of Tables 2,3,4 and 5 for a more detailed comparison of published works. More specifically, each table contains the mean age of the participants, gender distribution, stimuli and input used, methodology and conclusion. This allowed us to better situate our work in the literature, as well as highlight our work’s novelty.
2. We have added other performance comparisons with a prior approach that computed eye tracking variables and compared different machine learning models for comparison)^1,2^.
3. We have included a new section that provides a summary of different publicly available datasets for classification. This contains Table 1 that lists information about the reference, their target application area, the mean age of the participants, sample size, stimuli used and data format provided by the authors.

Minor changes:

1. We have revised the manuscript format to meet PLOS ONE’s style requirements.
2. We have provided more information about the gender distribution, eye-tracking procedures and clinical measures.
3. We have made improvements to the figures by providing more information in their corresponding captions.
4. Removal of the snapshot of the copyrighted stimuli.

Kind Regards,

The Authors

**Reviewer #1:**

The following are the major issues I found with the paper:
The paper is very hard to follow, it is very jumbled.

1. The paper is not laid out in a standard sections: Limitations and Results are before Methods. It is generally Methods, Results, Limitations. I did not understand why the sections were jumbled, made it difficult to understand the paper.

**RESPONSE:** Thank you for pointing this out. We have revised the manuscript to meet PLOS ONE’s style requirements.

2. References are very haphazard. Some citations have all the authors listed, some end in et al. Kindly stick to one of the two.

**RESPONSE:** Thank you for pointing this out. We are using a reference management software package and Google Scholar to pull out the bibliographic information. As a result, we did not have control over the way references were previously displayed.

3. No base line results. The paper provides no baseline results on their dataset from previous studies. A table that shows previous methods results and the paper's approach will be nice and will help catch the attention of the reader.
Previous methods reported a performance of 93.45% and the proposed approach is 94.47% without a comparison on same dataset following the same cross validation protocols, it is difficult to say if it was the method, the data or just a statistical anomaly.

**RESPONSE:** Due to the difficulty of finding the open-source implementations of the code/s used in the reviewed papers, it was hard to provide a results comparison. In addition, the published datasets are in a different format from what our model requires, making a straightforward comparison impossible. Initially, we used the simple thresholding approach^3-5^ implemented previously for comparison. Following your suggestion, we have also now followed the approach used by Canavan, et al. ^1,^Fabiano, et al. ^2^ for additional comparisons. The results are reported in a new section entitled ‘Comparison with other approaches’.

**Reviewer #2:**

Thank you for the opportunity to review the manuscript “Using visual attention estimation on videos for automated prediction of Autism Spectrum Disorder and symptom severity in preschool children”.
The study aimed to predict and stratify ASD using visual attention estimation. I commend the authors for their interest in autism and innovative computational models. I think this study could be interesting for the scientific community, particularly for the stratification model based on visual attention. However, I cannot recommend this manuscript for publication in its current form and I suggest major revisions. I hope that the following comments will assist the authors in their revision process.

**RESPONSE:** Thank you for acknowledging our work. We understand your concerns about the original manuscript and hope that we have sufficiently addressed them in our revision.

- In the introduction section, authors claim that “there is a lack of diagnostic paradigms that leverage dynamic stimuli” (line 68); they “go beyond static visual stimuli and utilize dynamic visual stimuli for eye tracking experiments” (lines 70-71), and their approach “provides an extension from static stimuli, widening the diagnostic paradigm to include dynamic stimuli” (lines 165-166). It seems that the use of dynamic visual stimuli in the computational identification of ASD is something novel that it is introduced here.

**RESPONSE:** We appreciate this request for clarification on the novelty claim about the use of dynamic stimuli. We revised the manuscript to make it clearer that the novelty of our approach is the use of saliency estimation on dynamic stimuli, rather than just the sole use of dynamic stimuli. It was evident in our original manuscript that there were studies that utilised dynamic stimuli for their experiment. However, most of them do not estimate visual saliency and instead use either raw eye-tracking data or computed eye-tracking variables (such as average fixation duration) and disregard the pixel information around the eye fixation. In fact, the most common approach was to convert the eye-tracking data into a scanpath image (i.e., the trace of eye movement) and perform image classification. This will be further clarified in the next responses.

- Nevertheless, there are previous instances of machine learning applications on visual attention towards dynamic stimuli (see the contribution of Minissi et al. (2022) in which four studies using dynamic stimuli are presented.<https://link.springer.com/article/10.1007/s10803-021-05106-5>), and authors used the same dynamic stimulus of Moore at al. (2018) in which a computational identification model of ASD is reported.

**RESPONSE:** Thank you for sharing an additional reference that is related to our work. We have reviewed it and added relevant references to the revised manuscript. Again, we believe that we were not clear in the original manuscript that the novelty of our approach was not just about using dynamic stimuli. We hope that the revised manuscript with the addition of tables (Tables 2,3,4 and 5) and better discussion sections provides more clarity about the differences between the existing approaches and ours. Maybe we are missing something, but we are not sure about the reference in Minissi, et al. ^6^ that used the same dynamic stimulus Pierce, et al. ^3,^Pierce, et al. ^4,^Moore, et al. ^5^. Could you please elaborate on this reference? The reference that we found that uses a similar stimuli was by Oliveira, et al. ^7^.

- In addition, in contrast to the idea that using dynamic stimuli is somehow “novel”, in line 122 authors described some literature using dynamic stimuli and visual attention for ASD classification; thus, which is the novelty of the present study regarding the type of stimulus? I suggest authors define clearly how this study differs from previous literature using the same type of social stimuli and which is the novelty.

**RESPONSE:** Thank you for pointing this out. As outlined in the table below, while there are studies that use dynamic stimuli, the most common approach was to convert the eye-tracking data of the participants into a scanpath image and perform classification. Another technique computed eye-tracking variables related to saccadic movement and use them as features for classification. On the other hand, our approach leverages spatiotemporal information learned by a dynamic saliency model and extracts features using the eye-tracking data of the participants for classification. This is the main difference between our approach and the published studies that utilise dynamic stimuli. To better highlight the differences and situate our research within the research landscape, we provided more discussion in the manuscript and added tables (Tables 2,3, 4 and 5) in each section of the related work section to provide more detailed information about the published research.

| Carette, et al. ^8^ | Video stimuli, eye-tracking variables related to saccadic movement of the eye. This includes saccadic amplitude, saccadic acceleration (average and maximum), saccadic deceleration (maximum), saccadic velocity (average and maximum), the rate of maximum velocity for a given saccade and the saccade duration. |
| --- | --- |
| Carette, et al. ^9^ | Video stimuli but eye tracking data was converted to an image which was then used for classification. |
| Elbattah, et al. ^10^ | Video stimuli but eye tracking data was converted to an image which was then used for classification. |
| Wan, et al. ^11^ | Video stimuli where a female actress was mouthing the alphabet |

- From lines 103 to 150, a brief state of the art regarding ASD classification based on static and dynamic stimuli is presented. It is essential to give an idea to the reader about previous literature. I suggest authors discuss these studies more deeply rather than just mention them. The relation between the present study and the previous ones needs to be clarified.

**RESPONSE:** This remark is related to the previous one. We agree that further clarification on similarities (use of dynamic stimuli) and differences (leveraging dynamic visual attention) between previous studies and ours will help us better situate our work in the current literature and highlight the novelty of our approach. Thank you for this suggestion. In addition to the new tables in the related work section, we have expanded the discussion in each section to address this clarification.

- Be careful with terms in the paragraph at line 151: the risk of ASD is not the same as ASD symptom severity. The comparison scores of the ADOS (used in the present study) give an idea of the severity of ASD instead of the risk of ASD, and in this paragraph, some studies assessing the risk of ASD are presented.

**RESPONSE:** Thank you for pointing it out as we completely missed this one. We have now revised that section to mention that there was a study about risk prediction, which is different from severity prediction. We also added some background information from the clinical literature that supports the possibility of using eye-tracking data for severity prediction.

- Line 194: it is surprising finding a comparison between the present model and those of Pierce et al. (2011) and Moore et al. (2018) since the authors should have reported in the introduction that this would have been a study purpose. I suggest the authors revise the introduction section to define study goals clearly.

**RESPONSE:** We agree that the comparison with Pierce, et al. ^3,^Pierce, et al. ^4,^Moore, et al. ^5^ was not properly introduced. The initial aim of this comparison was to provide a baseline performance since we did not have other models/methods and additional datasets to compare our results with. We have revised the manuscript to set the stage for later comparison. We also added another comparison with a previous approach^1,2^ for an additional baseline comparison. The results are reported in a new section entitled ‘Comparison with other approaches’.

- The discussion is short and leaves the reader to intend that using dynamic stimuli is a novelty of this study, but it is not. The discussion should present study results, their comparison with previous literature, and the step forward that the study made. How the current model improves ASD identification compared to previous models? How do the findings relate to Minissi et al. (2022) and Kollias et al. (2021) reviews?

**RESPONSE:** As mentioned above, we agree that the sole use of dynamic stimuli is not the novelty of the study. Instead, we leverage dynamic visual saliency detection to advance the literature. Previous research only use either raw eye-tracking data or computed eye-tracking variables without using the visual information around the fixation area. We have expanded the discussion to address the comments raised above. In particular, we provided a summary of the literature, highlighted the benefits of our approach, compared our results with previous models and provided some discussion about future work.

Regarding the improvements in ASD identification, it is difficult to deduce from our results since we were not able to compare our model with sophisticated models that use deep learning. Nevertheless, our approach outperforms a simple thresholding technique^3-5^ and handcrafted features combined with machine learning approaches^1,2^ in both ASD classification and symptom severity prediction.

- We could say that unequal groups and reduced sample sizes are the major limitations of this study, and findings should be interpreted cautiously.

**RESPONSE:** We are aware that the unequal groups and small sample sizes are the major limitations of this study. It was a bit unfortunate that we were not able to recruit TD children with a sample size comparable to our ASD group. Nevertheless, we made our best effort to provide detailed limitations of our study and were careful to make strong claims about the results of our study. More specifically, we noted in the original manuscript:

“While our results are promising, our approach needs to be trained and tested on a much larger dataset before it can be utilised in clinical settings.”

And added:

“Extensive experiments are necessary before the true clinical utility and usability of our proposed method can be realised.”

- In line 269 authors claimed that the LOOCV approach is suitable for their model and addresses the group imbalance. However, for my little knowledge of data science, variant of k-fold cross-validation are recommended for evaluating the performance of a model when groups are imbalanced, and the sample size are reduced. Stratified k-fold cross-validation ensures that each fold of the cross-validation process has roughly the same proportion of samples from each group as the original dataset. This approach is particularly useful when dealing with imbalanced datasets or unequal group sizes.

**RESPONSE:** Given the limitations of our study, we agree that rigorous model validation and evaluation are crucial. Just to clarify, LOOCV is an extreme case of k-fold cross-validation. That is, LOOCV is the same as k-fold cross-validation when k is equal to the number of samples in the dataset. More information about this will be provided below.

- In contrast, LOOCV may not be suitable for small datasets with unequal group sizes because it requires leaving out a single data point at each iteration, which can result in too few data points in the training set, making it difficult to obtain a reliable estimate of the model performance.

**RESPONSE:** While the first statement is correct, LOOCV does not result in too few data points in the training set since only a single data point is removed every iteration. This singled-out data point is used for testing, while the remaining data points are used for training. In the k-fold cross-validation scenario, the number of data points to be removed and used for testing is equal to the number of samples/k, resulting in less training data. As a result, we have more data points in the training set compared to the data points gathered in k-fold cross-validation. Our LOOCV approach is valid and it has been adopted by previous research^12-17^.

- Therefore, in this scenario, stratified k-fold cross-validation can be a good option as it strikes a balance between the computational efficiency of k-fold cross-validation and the need to account for group imbalances in the dataset. As reported in line 270, a similar study used 5-fold cross-validation to address the class imbalance. Still, the authors should have discussed why they used LOOCV compared to other strategies.

**RESPONSE:** Thank you for pointing this out. Due to our small dataset, we decided to use LOOCV. If we were to use a stratified k-fold cross-validation strategy, there will be fewer examples in each fold and may not allow our model to learn better. While we mentioned that a similar study has used a 5-fold cross-validation strategy, their dataset is much bigger than ours (almost double ours). We agree that the stratified k-fold cross-validation strategy accounts for group imbalances since it ensures that each fold has the same class distribution. However, in the case of LOOCV, removing 1 sample from the train split to use as a test sample also does not drastically change the class distribution. The combination of being able to use as much training data as possible while also maintaining similar class distribution was the reason why we used LOOCV. We highlight in our revised manuscript that several papers adopted this strategy in ASD classification. We revised the manuscript to provide more discussion on why we used LOOCV compared to other strategies.

- Lines 318-322: Please deepen the description of how the experimenter calibrated the eye tracking system with young children (4 yo) with high-moderate severity of ASD.

**RESPONSE:** Thank you for this suggestion. We revised the manuscript to reflect this. More specifically:

“The eye-tracking procedure was conducted during a clinical assessment or the intake assessment for entry to an early intervention program. Multiple attempts were made to ensure that the eye tracker has been calibrated properly for accurate data collection. Multiple attempts were also made to ensure that the participants were engaged during the experiment. As a result, depending on the capacity of the child, the procedure was conducted over 2 to 3 sittings or with smaller breaks in between. The overall clinical assessment and eye-tracking procedure were completed in approximately 2.5h per participant.”

Minor comments
- In the result section, if I got it right, figure 1 and 2 the model metrics are presented as the number of fixations increased, but in the horizontal axis of both plots “fixation length” is reported.

**RESPONSE:** Thank you for pointing this out. In this work, the number of fixations is the same as the fixation length.

- How symptom severity has been measured should be reported in the participant section. Some information is unclear and presented in lines 411-413.

**RESPONSE:** Could you please clarify what you meant by this? We are not quite sure how to discuss the quantification of symptom severity other than by looking at the participant’s ADOS comparison scores, which is the ADOS calibrated severity score (CSS) of the participants in our study. We hope that the use of the term CSS instead of comparison scores clarifies this query.

- In the limitation section, it is reported that there was a gender imbalance in the sample; however, this is not reported in the participant section.

**RESPONSE:** Thank you for pointing this out. The participant section now includes the gender of both the ASD and TD groups. More specifically, there were 9 females in the ASD group and 9 females in the TD group. As mentioned in the limitations section, our ASD group is skewed towards males.

- In addition, the limitation section is long, and some of the info may be moved to further sections of the paper.

**RESPONSE:** Thank you for the suggestion. We revised the manuscript to address this.

**Reviewer #3:**

The authors present a machine-learning-based model for automatic screening of ASD and for severity (two levels) prediction, which considers features extracted from dynamic visual stimuli. The reported experimental results show promising performance and comparable to the performance reported by related works on static stimuli.

- This paper addresses a very relevant and timely topic, which, although a is focus of a very active research work lately, still requires more studies, models and datasets. Therefore, I think that the paper can provide useful insights for the research community in the field.

**RESPONSE:** We are happy to see that the reviewer thinks our research can contribute to the growing literature on using eye-tracking data for ASD classification.

- However, I'm concerned about the limited validation of the proposed approach, which I'm not sur if it is sufficient for a journal publication. In this sense, my major concern is that the proposed approach has only been validated using a leave-one-out technique. Although it is a common and valid practice, I would expect a deeper validation of models addressed to applications like this, based on the use of separate training and testing datasets. Several works have reported a significant decrease of the performance of the models when using a "secret" testing dataset in comparison with leave-one-out methods. I'm aware of the difficulty of doing this, given the lack of publicly available datasets, but probably the authors can report some more results splitting their dataset in two subsets.

**RESPONSE:** As evident in our related work in the original manuscript, we tried our best to review the literature for any publicly available datasets. Although there were a few, our approach is not directly applicable due to the nature of the released data. More information is provided in the next response. We acknowledge that we did not clarify this issue in the original manuscript. We now provide a discussion on this in the revised manuscript. Concerning splitting our dataset into two subsets, we think that it will cause too few sample sizes, given that our original dataset is already considered small. We agree that this is not ideal, but this is a primary limitation of our study. Nevertheless, we made our best effort to provide detailed limitations of our study and were careful to make strong claims about the results of our study.

- In addition, the proposed approach has been compared with a simple thresholding approach [10-12], but they have not compared their model with any pf the existing ones for dynamic stimuli. I'm also aware of the lack of open-source models, but in case the authors have identified any, it would be nice to compare the performance of their approach with those.

**RESPONSE:** As will be discussed in the next response, a straightforward comparison of our model with other models for dynamic stimuli is not possible because the inputs are different. First, the most common approach, as shown in the new Table 4, is to convert the eye-tracking data into an image and perform image classification. On the other hand, our approach requires the stimuli and the associated eye-tracking data of the participant for classification.

- In this sense, if the authors have identified any publicly available dataset (e.g., Carette and colleagues have recently published one) it would be nice if they can use it to further test their model. If not, at least, I think that a review of existing datasets (probably as part of the related work section) would be beneficial to the readers.

**RESPONSE:** We agree that the evaluation of our model in a different dataset is important. However, this is challenging given the nature of the current publicly available dataset. For example, Carette, et al. ^18^ released the visualisation of the eye-tracking data of the participants as images, but not the stimuli and the associated eye-tracking data of the participants. Since our model utilises both of the latter data, direct application of our model on the Carette, et al. ^18^ data is not possible. We appreciate the suggestion to provide a review of existing datasets and agree that it would be a helpful resource for readers. As a result, we have added an entire section about existing publicly available datasets (Table 1). In addition, our related work includes tables that contain the datasets used by the reviewed work. This allows for easier (possible) comparison with other approaches.

Some other comments are:
- In line 162-163, the authors say "limited research has been performed to explore the effectiveness of temporal information captured during dynamic stimuli viewing". It would be nice to elaborate on this and provide more details about what do the authors mean with it. I would expect from almost all the related works mentioned by the authors working with dynamic stimuli to exploit the temporal information that this stimuli provide (in contrast to using static ones).

**RESPONSE:** We agree that our original manuscript was not clear about the novelty of our approach because we did not provide elaboration on the techniques used by previous works. We hope that the new tables (Tables 2,3,4 and 5) in the related work section and further discussion clearly highlight the differences in techniques used by previous works and our paper. For example, while all studies in the Eye-tracking on Dynamic Stumili for ASD diagnosis section utilise dynamic stimuli, the most common approach was to visualise the eye-tracking data as an RGB/grayscale image of the scanpath (i.e., the trace of the eye movement) and perform ASD classification. While spatiotemporal information may have been encoded in this representation, this approach may have potentially lost some important information, such as the sequential nature of fixations.

- I may have missed it, but I have not seen the specific numbers related to the gender of the participants in the test (although the authors mentioned that the pool of participants was unbalanced).

**RESPONSE:** Thank you for pointing this out. The participant section now includes the gender of both the ASD and TD groups. More specifically, there were 9 females in the ASD group and 9 females in the TD group. As mentioned in the limitations section, our ASD group is skewed towards males.

- Also in this sense, although it was mentioned as a limitation by the authors, the unbalance of the dataset in terms of children with ASD and with TD is an important flaw.

**RESPONSE:** We agree that this is an important flaw. Nevertheless, we made our best effort to provide detailed limitations of our study and were careful to make strong claims about the results of our study. More specifically, we noted in the original manuscript:

While our results are promising, our approach needs to be trained and tested on a much larger dataset before it can be utilised in clinical settings.

And added:

“Extensive experiments are necessary before the true clinical utility and usability of our proposed method can be realised.”

- In terms of the stimuli used, although the detail information can be found in the papers [10-11], for self-contention of this paper, it would be nice that the authors provide more info of them. For example, which was the duration of the scenes? How were they presented to the participants (e.g., in random order)? Were the videos played in full-screen? Which was the resolution of the videos?...

**RESPONSE:** Thank you for pointing this out. We updated the manuscript to include more information about the dynamic stimuli. More specifically, the stimulus contained a total of 28 different scenes and was presented in order, based on the original published stimulus. It has a resolution of 1281 x 720 pixels and contains a total of 1,488 frames, which is equivalent to 61 seconds of playback. The stimulus was played on full-screen.

- In line 335, the authors say that they have used "An additional data quality assessment... to determine the overall nature of the visual attention of the participants to the stimuli", but no specific information has been provided about it.

**RESPONSE:** The sentence following the quoted statement above **(Line 318)** provides specific information about the visual attention between groups. More specifically, we found no significant difference in visual attention between groups.

- In line 339, the authors state that "An independent-samples t-test was used to investigate differences in visual attention across two groups for diagnosis (ASD vs. TD) and severity prediction (moderate vs. severe)". However, no results are reported or no indications on were to finde them are included.

**RESPONSE:** We have now removed this statement because it is a repetition of the statement in Line 318.

- In the expression in line 375, how the coefficients (0.1) that apply to L_CC and L_NSS were obtained?

**RESPONSE:** We followed the original implementation of the saliency detection model^19^ for the weighting (coefficients) of the loss functions. We updated the manuscript to clarify this.

Finally, the paper is, in general, well written, but a careful proof-read would be beneficial to avoid some typos and minor errors. For example, in the introduction, line 59 "Pierce et al." appears twice; line 104 "to an autism diagnosis" may be "to autism diagnosis"; please check for extra or missing spaces before or after commas; in lines 276 and 277 authors use "comorbid" and "co-morbid"; etc.

**RESPONSE:** We appreciate the reviewer’s comment about some typos and minor errors. We have proofread and revised the manuscript to address these.

References

1	Canavan, S. *et al.* Combining gaze and demographic feature descriptors for autism classification in *2017 IEEE International Conference on Image Processing (ICIP).* 3750-3754 (IEEE) (Year).

2	Fabiano, D., Canavan, S., Agazzi, H., Hinduja, S. & Goldgof, D. Gaze-based classification of autism spectrum disorder. *Pattern Recognition Letters* **135**, 204-212 (2020).

3	Pierce, K., Conant, D., Hazin, R., Stoner, R. & Desmond, J. Preference for Geometric Patterns Early in Life as a Risk Factor for Autism. *Archives of General Psychiatry* **68**, 101-109, doi:10.1001/archgenpsychiatry.2010.113 (2011).

4	Pierce, K. *et al.* Eye Tracking Reveals Abnormal Visual Preference for Geometric Images as an Early Biomarker of an Autism Spectrum Disorder Subtype Associated With Increased Symptom Severity. *Biological Psychiatry* **79**, 657-666, doi:<https://doi.org/10.1016/j.biopsych.2015.03.032> (2016).

5	Moore, A. *et al.* The geometric preference subtype in ASD: identifying a consistent, early-emerging phenomenon through eye tracking. *Molecular autism* **9**, 1-13 (2018).

6	Minissi, M. E., Chicchi Giglioli, I. A., Mantovani, F. & Alcaniz Raya, M. Assessment of the autism spectrum disorder based on machine learning and social visual attention: A systematic review. *Journal of Autism and Developmental Disorders* **52**, 2187-2202 (2022).

7	Oliveira, J. S. *et al.* Computer-aided autism diagnosis based on visual attention models using eye tracking. *Scientific reports* **11**, 1-11 (2021).

8	Carette, R. *et al.* Automatic autism spectrum disorder detection thanks to eye-tracking and neural network-based approach in *Internet of Things (IoT) Technologies for HealthCare: 4th International Conference, HealthyIoT 2017, Angers, France, October 24-25, 2017, Proceedings 4.* 75-81 (Springer) (Year).

9	Carette, R. *et al.* Learning to Predict Autism Spectrum Disorder based on the Visual Patterns of Eye-tracking Scanpaths in *HEALTHINF.* 103-112 (Year).

10	Elbattah, M., Carette, R., Dequen, G., Guérin, J.-L. & Cilia, F. Learning clusters in autism spectrum disorder: Image-based clustering of eye-tracking scanpaths with deep autoencoder in *2019 41st Annual international conference of the IEEE engineering in medicine and biology society (EMBC).* 1417-1420 (IEEE) (Year).

11	Wan, G. *et al.* Applying eye tracking to identify autism spectrum disorder in children. *Journal of autism and developmental disorders* **49**, 209-215 (2019).

12	de Belen, R. A. J., Bednarz, T. & Sowmya, A. EyeXplain Autism: Interactive System for Eye Tracking Data Analysis and Deep Neural Network Interpretation for Autism Spectrum Disorder Diagnosis in *Extended Abstracts of the 2021 CHI Conference on Human Factors in Computing Systems.* Article 364 (Association for Computing Machinery), doi:<https://doi.org/10.1145/3411763.3451784> (Year).

13	Liu, W., Li, M. & Yi, L. Identifying children with autism spectrum disorder based on their face processing abnormality: A machine learning framework. *Autism Research* **9**, 888-898, doi:10.1002/aur.1615 (2016).

14	Jiang, M. & Zhao, Q. Learning Visual Attention to Identify People with Autism Spectrum Disorder in *2017 IEEE International Conference on Computer Vision (ICCV).* 3287-3296, doi:10.1109/ICCV.2017.354 (Year).

15	Chen, S. & Zhao, Q. Attention-Based Autism Spectrum Disorder Screening With Privileged Modality in *2019 IEEE/CVF International Conference on Computer Vision (ICCV).* 1181-1190, doi:10.1109/ICCV.2019.00127 (Year).

16	Zhao, Z. *et al.* Classification of Children With Autism and Typical Development Using Eye-Tracking Data From Face-to-Face Conversations: Machine Learning Model Development and Performance Evaluation. *J Med Internet Res* **23**, e29328, doi:10.2196/29328 (2021).

17	Jiang, M. *et al.* Classifying individuals with ASD through facial emotion recognition and eye-tracking in *2019 41st Annual International Conference of the IEEE Engineering in Medicine and Biology Society (EMBC).* 6063-6068 (IEEE) (Year).

18	Carette, R., Elbattah, M., Dequen, G., Guérin, J.-L. & Cilia, F. Visualization of eye-tracking patterns in autism spectrum disorder: method and dataset in *2018 Thirteenth International Conference on Digital Information Management (ICDIM).* 248-253 (IEEE) (Year).

19	Wang, W., Shen, J., Guo, F., Cheng, M.-M. & Borji, A. Revisiting video saliency: A large-scale benchmark and a new model in *Proceedings of the IEEE Conference on Computer Vision and Pattern Recognition.* 4894-4903 (Year).
